# Basic Process Equation for Analytical Chemistry – An Inclusive and Conciliatory Approach

**DOI:** 10.1021/acs.analchem.5c05444

**Published:** 2025-12-30

**Authors:** Luis Cuadros-Rodríguez

**Affiliations:** Department of Analytical Chemistry, 117396 University of Granada, C/Fuentenueva s/n, Granada E-18071, Spain

## Abstract

The proposal outlined in this article aims to use information flow as a benchmark for analytical chemistry. It starts with raw information, mostly implicit and nonobvious (latent information), enclosed in the analytical signal, which is transformed into explicit and interpretable target chemical information (patent information). A basic process equation is proposed, derived from the currently accepted definition of analytical chemistry, which reflects the paradigm change from measurement to information. The main innovation lies in the inclusion of a term called the 'analytical operator,’ which is responsible for transforming the information. The terms of this equation are interpreted and matched with different analytical methods, as well as with the R&D activities involved in current analytical chemistry. The proposal provides, for the first time, an inclusive approach that reconciles traditional analytical methods, relying on univariate data, with analytical methods derived from the application of chemometrics (or, in the same way, data mining, machine/deep learning, artificial intelligence, etc.) based on multivariate data. For better understanding, the determination of the iodine value in vegetable oils has been considered as a guiding example demonstrating how the proposed process equation is valid throughout the different periods of analytical chemistry, regardless of the analytical method applied.

## Graphical abstract

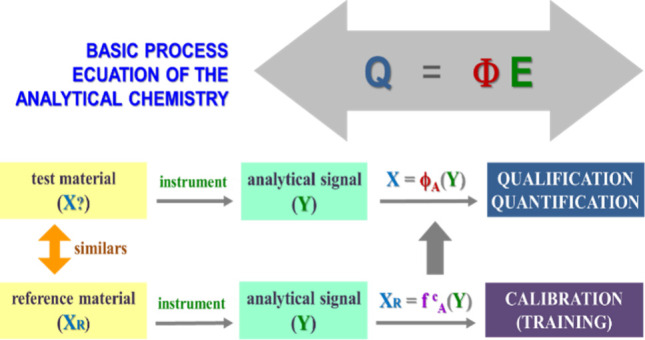

## Introduction

Analytical chemistry emerged at around the same time that chemistry became established as a science independent of physics and other natural sciences in the 17th century. However, it was not until a century later that it became established as an independent discipline within chemistry, with its own identity. From the 20th century onward, with the widespread introduction of analytical instruments focused on measuring properties that are generally physical in nature, its place has been a constant subject of debate, and indeed remains so today.^1^ In this story of encounters and disappointments, the period spanning the last two decades of the last century (1980–2000) is particularly noteworthy when the scientific nature of analytical chemistry was called into question, giving rise to considerable controversy among chemists. Diving into the literature of the time, it is easy to find scientific articles arguing in favor of both positions, which are in principle opposed: reaffirming the scientific content of analytical chemistry^2,3^ and, conversely, describing analytical chemistry as a technology or methodology supporting chemistry and other sciences.^4,5^ Furthermore, to confuse matters further, at that time the metrological aspect of analytical chemistry was also revindicated^6^ and the term 'analytical science’ was proposed as a substitute for analytical chemistry.^7^ All of this led to the coexistence of multiple personal opinions and definitions of analytical chemistry,^8^ not all of which were consistent with each other. Some of them still remain, which has resulted in increased confusion.

In an attempt to settle the debate and find the place of analytical chemistry in science, Fresenius’ Journal of Analytical Chemistry (the precursor to the current Analytical and Bioanalytical Chemistry) launched a competition in 1991 to propose a definition and interpretation of analytical chemistry, which received 21 proposals from chemists in 13 countries. The results were published in the same journal^9^ and, as a consequence, the Working Party of Analytical Chemistry (WPAC) of the Federation of European Chemical Societies (FECS), meeting in Edinburgh (United Kingdom) in 1993, established that analytical chemistry is the *scientific discipline that develops and applies strategies, instruments, and procedures to obtain information on the composition and nature of matter in space and time*.^10^

This definition, is still in force and has recently been included in the IUPAC Compendium of Terminology in Analytical Chemistry, published in 2023.^11^ This entails a change in the main focus of analytical chemistry, which was centered on measurement and is now focused toward information; it is significant here the terms 'chemistry’ and 'measurement’ do not even appear in the definition, and are restricted solely to the concept of quantitative analysis.^11^

In this author’s opinion, the decision to downplay the measurement was highly appropriate, since analytical chemistry should not be defined by the tools or methodologies involved. These may be of any nature and belong to other scientific disciplines in addition to those specific to chemistry, e.g., physics, biochemistry, geochemistry, chemical engineering, materials science, food science, environmental science, restoration of cultural heritage, and many more. Indeed, this wrong view, when applied to teaching analytical chemistry, has done (and continues to do) a lot of harm, as it leads students to think the fundamentals of analytical chemistry are derived from those disciplines.^12^ Even today, this misconception persists when recent advances in analytical chemistry, such as the use of microfluidic devices or nanomaterials, are presented as mere applications subject to an extensive theoretical introduction on the fundamentals of such systems.

The importance of information was highlighted early on by Kowalski, who in 1981 published a paper defending the approach of analytical chemistry as an information science, based on chemometrics.^13^ However, Kowalski’s pioneering spirit drove him further, and in 1994 he published a visionary article entitled 'Theory of Analytical Chemistry’ in which, through 39 equations based on mathematical tensor algebra, he described what should constitute the analytical corpus.^14^ He argued analytical chemistry should have its own laws and theories that confer identity upon it. In fact, the Kowalski’s theory covers the types of data and calibrations and establishes the rules for determining what information can be mined from the data provided by any analytical instrument or method. However, the analytical community was not yet ready to assume such concepts, and the proposal went largely unnoticed, except by chemometricians. Nevertheless, this article defends the validity of the contents of the aforementioned paper and the postulates that make up the theory.

Analytical chemistry has evolved notably since its early days as a modern science in the 18th century. From a broad historical perspective, three periods or ages could be distinguished. The transition from one to the next marked by the introduction of a particular scientific (r)­evolution.^15,16^ The first period begins with wet analytical chemistry (the chemical age). It is based mainly on chemical reactions involving solids, solutions, and gases. It is the time of test tubes, balances, volumetric equipment and gas collectors discoveries were mainly based on visual observation of the results. The second period is caused by the implementation of analytical instrumentation. It promotes analytical processes to focus mainly (though not exclusively) on physical phenomena, whereby measurement acquires the status of being considered the most significant stage and the whole analytical process gravitates around it (the metrological age). The third period comes with the advent of informatics and computerized systems (the data science age). It enabled automated control of instrumentation and, above all, facilitated the extensive performance of complex mathematical calculations.^17^

While the data science age is not fully consolidated yet, a new (r)­evolution is emerging, still in development, based on the use of artificial intelligence (AI). It is expected that in its most advanced stages, yet to be developed, it will not require mathematics based on more or less complex numerical operations. This idea is best explained with an example. Humans are able to differentiate between two photographed faces almost instantly, even if they look quite similar, simply by looking at both images. To do this, we do not apply numerical calculations, i.e., we do not look for numerical markers or apply any similarity algorithms, but rather the differentiation is based on a holistic visual assessment of the respective images. Likewise, AI could qualify or quantify an analytical target on two similar samples of the same material, showing some distinctive feature, just by evaluating selected multidimensional instrumental signals (or images) with no need to process large set of numerical data. This would be the artificial intelligence age, in which analytical devices would emulate the human brain ability for imagery processing. The concerned analytical technique could be referred to as 'imagimetrics.' They would operate without mathematics, at least in the form we currently know it, which is not yet developed. Figure 1 outlines the major features of the different (r)­evolutions in analytical chemistry.

**Figure 1:**
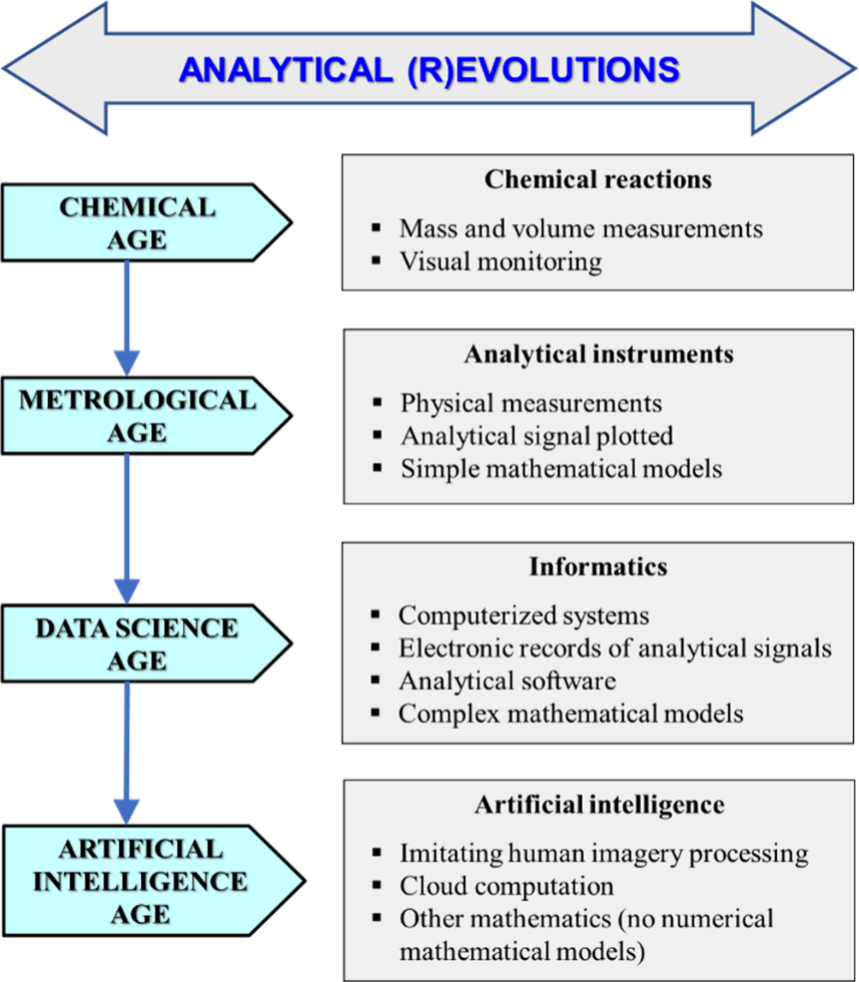
Main ages of analytical chemistry, with some of their specificities, which determine the historical scientific–technical (r)­evolutions involved.

Thus, periods focused on chemistry alone (the chemical age), physics (the metrological age), and mathematics and informatics (the data science age) and intelligence artificial (intelligence artificial age) are considered, all aimed at obtaining chemical information about material systems, bearing in mind that each one integrates the previous ones. In fact, chemical reactions are still present in many analytical processes, although as a result of the rise of Green Analytical Chemistry (GAC),^18^ the aim is to minimize their use or, ideally, avoid them altogether. However, measurement continues to play a role, since the usual way of accessing chemical information is through the obtention of an instrumental signal. Although from a strict point of view, most of the analytical signals are acquired and recorded in arbitrary units, so they do not fully meet the requirements of a metrological process.^19^

Chemometrics and, more recently, data mining and machine/deep learning (which share objectives and methodologies with the former) have entered modern analytical chemistry. And finally, the incipient artificial intelligence, which is yet to show its full potential. They are here to stay, much to the dismay of some conservative analytical sectors usually located outside the industry, and linked above all to certain traditional academic areas and those related to government (e.g., official food control).

Today’s analytical chemist must learn to combine each of these three approaches, depending on the analytical problem to be solved, and must not remain static in the face of improvements provided by supplier instrumentation companies. Particularly, the implementing of the advantages of the third (r)­evolution no longer be overlooked, as it involves the development of predictive models, beyond the passive use of software commanding the analytical instrumentation. The information flow should be the core of analytical chemistry, from the acquisition of raw data, through mining for useful information, to the transformation of raw data into relevant target information. This involves a deep rethinking of the analytical process.

## Single Process Equation for All Analytical Chemistry

As a result of the Edinburgh definition, the main objective of analytical chemistry would be stated as developing and applying scientific and technical instruments and methodologies of to obtain reliable and high-quality chemical information about a material system. This is (usually) not accessible through direct measurement, from related information embedded implicitly in a signal and/or analytical data obtained through experimental measurement. If target chemical information is symbolized by **Q**, and raw experimental information acquired and enclosed in a signal or data is symbolized by **E**, this objective can be expressed by the process (eq 1):

Q=ΦE 1

where **Φ** means the analytical operator, responsible to transform raw information **E** (which acts as the operand) into target chemical information **Q** (which is identified with the result of such operation). Note that eq 1 does not express a mathematical equation, but rather describes a process in accordance with the flow of information. This expression was already intuited by Currie in 1995,^20^ who even used the term operator with a similar meaning. Equation 1 deserves to be referred to as the 'basic process equation of analytical chemistry’ since its three terms cover the entire analytical methodology.

Indeed, the target chemical information **Q** reflects the feature of the material system from which information is intended to be obtained: an individual analyte, a family or groups of chemically related analytes considered as a whole, or an entire material that, when mixed with others, composes the material system under study. It also considers chemical proficiency indices and/or method-defined quality parameters. In addition, **Q** also involves the tier of the target information to be determined through analysis: qualitative (detection, identification, typification) or quantitative (quantification, distribution). A more detailed description of these issues can be found in the reference.^21^

In parallel, the term **E** refers to the type of data obtained from an analytical signal acquired experimentally after a measurement process. The analytical signal must be selected so that it captures the maximum useful information in the raw logging **E** which may be evident to the practitioner’s senses or, more commonly, be hidden in a nonevident way. Analytical signals are acquired from the response of the experimental system to a disturbance, which triggers a process of any nature (physical, chemical, or biological). From the analytical signal, the analytical data required to mine the target information **Q** is obtained. The analytical signal takes one of two ways: (i) it reveals a change that can be perceived by the senses (e.g., a color change typical of colorimetric tests), or (ii) it provides a set of numerical values (data). In the latter case, for a univariate analytical method, the data is represented by a single number. In contrast, in a multivariate analytical method, a set of representative numerical values is obtained from each signal, which may be of different dimensionality.^22^

Concerning the analytical operator **Φ**, it brings together all the operations necessary converting the raw information in the target information. Note that these operations are of all kinds and may refer to physical processes (e.g., colorimetric kits) or be based on mathematical operations; the latter issue will be discussed in detail later. In cases where raw information **E** is not evident, the conversion is preceded by a preliminary stage of tracing and mining critical (or useful) information. The analytical operator **Φ** is not unique, but depends on the material system under study; the type of analytical signal acquired and the nature of the raw information involved; the dimensionality of the analytical data obtained from the signal; and the tier of the intended target information.

Figure 2 shows the relationships between the information flow chain and the terms of the basic process equation. Once the material system is disturbed and a response is generated, the raw experimental information is acquired as an instrumental signal consisting of a data set. The analytical operator transforms the data into results. The analytical target information is identified with each result which, when interpreted, gives rise the intended knowledge.

**Figure 2:**
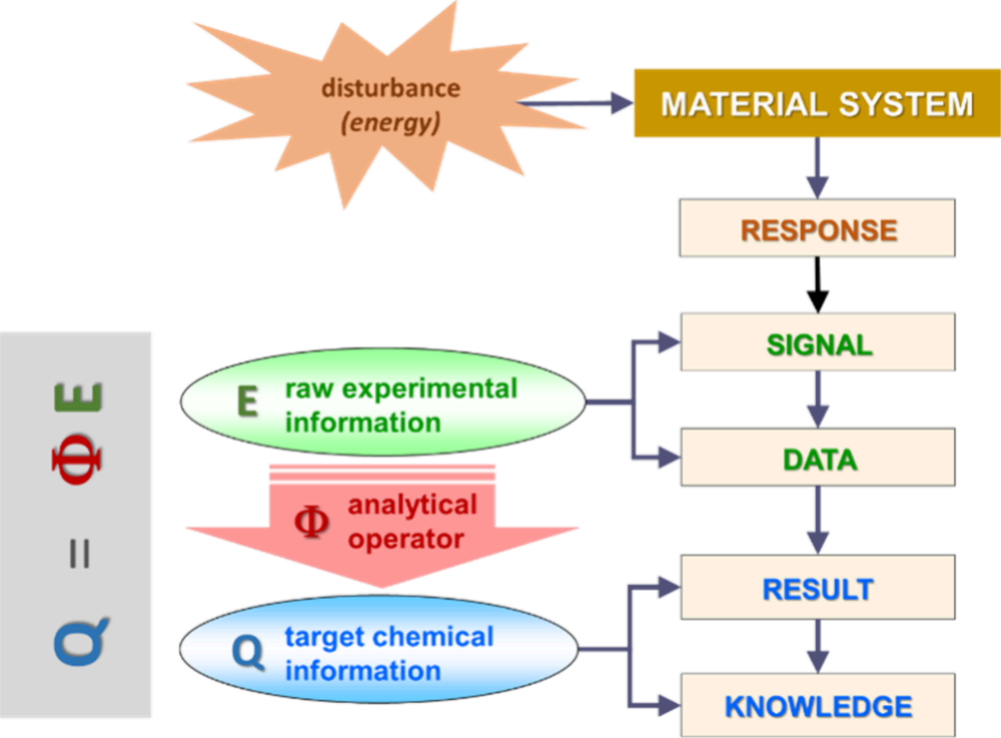
Relationship between the analytical information flow chain and the terms of the basic process equation of the analytical chemistry.

### Alternative Approach to the Analytical Process

It is common to find a flowchart showing the analytical process as a set of three (or more) steps focused on the measurement, which considers the steps before, during, and after the measurement. A simplified example of this measurement-focused scheme is shown in Figure 3A. However, this scheme has become obsolete since the current definition of analytical chemistry was recognized and accepted. It should have been replaced by an alternative based on information flow. Figure 3B shows a proposal based on three steps matching the terms of the basic process equation. The three tiers outlining the scheme are consistent with the three terms of the equation. The experimental step of acquiring raw information (nonexplicit/nonevident latent information), the step of converting or transforming the information, and the step of obtaining the target information intended (explicit patent information).

**Figure 3:**
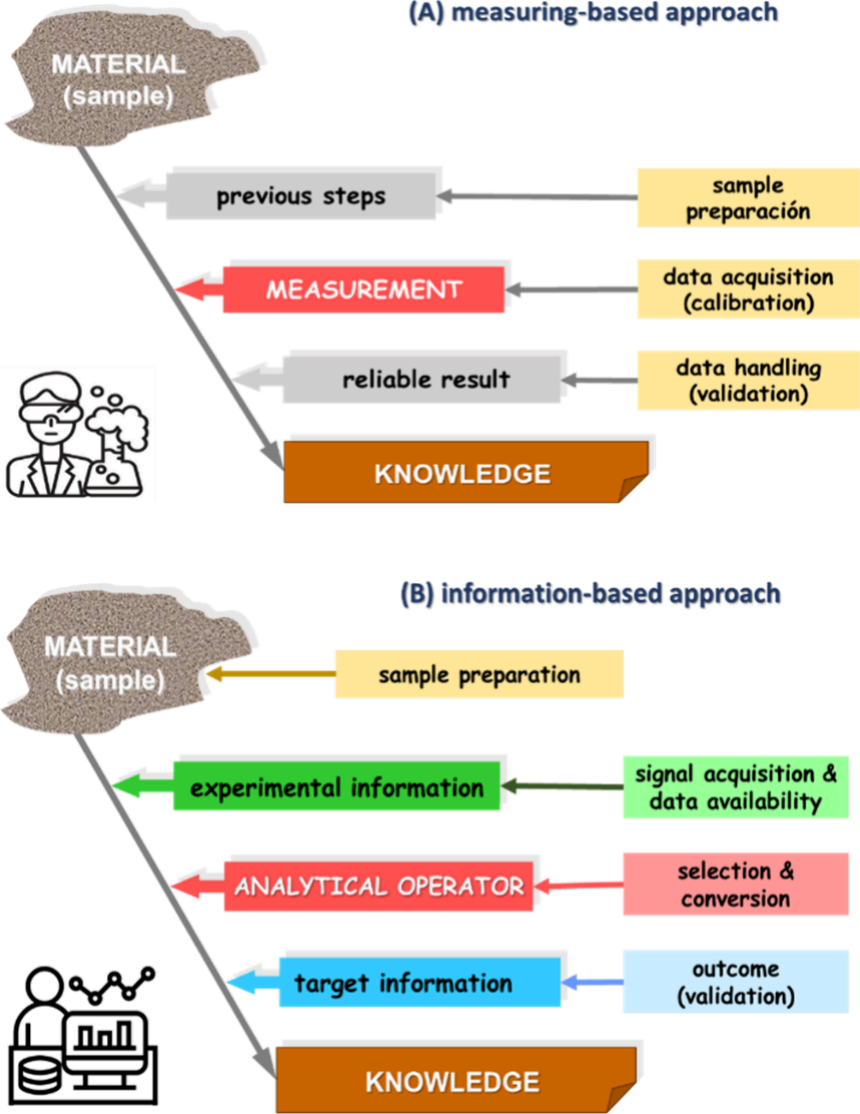
Analytical process: (A) outdated conventional measuring-based approach; (B) new updated information-based approach.

### Mathematical Formulation of the Basic Process Equation

In the current mathematical formalism, the information **Q** and **E** is replaced by numerical variables **X** and **Y**, and the analytical operator **Φ** adopts the mathematical function **ϕ**_**A**_, termed ‘analytical function’. Note that, as mentioned above, the setting up of artificial intelligence systems could change this classic mathematical approach. But while that happens, eq 2 expresses this mathematical approach.

$$X=\varphi_{A}(Y)[Y\overset{\varphi_{A}}{\to }X]$$ 2

**X**-variable constitutes the analytical target and, depending on the type of analytical method (qualitative or quantitative), will represent a quality (presence/absence, positive or negative identification, belonging or not to a certain category or class, etc.) or a quantity (a concentration, a value of a chemical proficiency index and/or a method-defined quality parameter). It is usually specified by a scalar (i.e., a single numerical value).

**Y**-variable consists of a set of numerical data derived from the analytical signal and is tensor in nature, which determines the analytical strategy to be applied.^23^ For univariate analytical methods, the **Y**-variable will be a scalar (zero-order tensor), while multivariate analytical methods are developed from data vectors (first-order tensor), data matrices (second-order tensor), or data parallelepipeds (third-order tensor); higher dimensionality is unusual, although feasible.^24^ However, even if the original **Y**-variable has an order greater than zero, it is common practice to reduce the dimensionality in order to obtain a scalar value representative of the entire signal, with the aim of performing a univariate method. This inevitably involves a loss of some of the raw experimental information and, unless the signal is specific (or highly selective) to the analytical target, the results could be biased.

The analytical function $\varphi_{A}$ establishes the functional relationship between both **X** and **Y** variables, and is specific to the target-analytical signal pair. Its inverse function is called 'physicochemical function’ $\varphi_{\mathrm{FQ}}$ and is given by the eq 3.

$$Y=\varphi_{\mathrm{FQ}}(X)[X\overset{\varphi_{\mathrm{FQ}}}{\to }Y]$$ 3

Note that each can be obtained by inverting the other: $\varphi_{A}$ **=** ${\varphi_{\mathrm{FQ}}}^{‐1}$ , or $\varphi_{\mathrm{FQ}}$ **=**. ${\varphi_{A}}^{‐1}$ From a chemical point of view, the physicochemical function has a better meaning since it establishes a relationship between the cause (material property, composition) and the effect (response to a physicochemical phenomenon and signal). However, from an analytical point of view, it is much more interesting to know the analytical function that enables the value of the analytical target to be determined from the data collected in the experimentally acquired analytical signal.

The analytical function (one for each analytical target) is not unique for each material system, but depends on the nature and features of the acquired signal, i.e., the performance of the instrument (and instrumental conditions), the characteristics and composition of the test portion to be measured (reagents, solvents, etc.), and, by extension, the material system under study (physical state, particular composition of the analytical matrix). The simplest analytical function to apply is the ‘identity function’: **X = Y**. These are not very common and are only used in univariate methods to obtain the value of some chemical proficiency indices and/or method-defined quality parameters (e.g., the octane rating of gasoline or the Rancimat stability of vegetable oil).

### Empirical Calibration Function

In some specific cases, the physicochemical function is known in advance from the application of a scientific rule or law: stoichiometry of the chemical reaction, Lambert–Beer law, Nerst equation, Coulomb’s law, Ilkovic equation, uniformity of isotopic ratios, etc. However, the methodology generally applied involves the *ad hoc* development of a representative empirical function. It is fitted via a regression method which involves representative reference materials (or chemical standards), i.e., materials with a well-characterized value of the **X**-variable. Note that the empirical function developed is a mathematical function that emulates the analytical function (or the physicochemical function), even though mathematically it has a different morphology. It is intended to show similar behavior in the working range of the analytical target values covered by the reference materials used. In practice, the actual analytical function is unknown and is not experimentally accessible in all its terms.

The empirical function, as well as the process leading to obtaining it, has different names depending on the type of analytical method. In a univariate strategy, the conventional names ‘calibration function’ and ‘analytical calibration’ are used, respectively. In this scenario, the physicochemical function is generally pursued, and by inverting it, the analytical function is then obtained. For multivariable methods, they are referred to as ‘models’ and ‘training or analytical modeling’ and usually aim to directly emulate the analytical function. Whatever name is used, the analytical interpretation is equivalent.

The calibration function **f** ^**c**^ (as either a univariate calibration function or a multivariate model) is mathematically represented in eq 4. Note that the subscript 'R’ symbolizes data based on reference materials.

$$X_{R}=f^{c}(Y)[Y\overset{f^{c}}{\to }X_{R}]\Rightarrow \varphi_{A}≡f^{c}$$ 4

The meaning of the **X** and **Y** variables is usually reversed in multivariate analytical methods, where **X** is used to represent the experimental data and **Y** to symbolize the analytical target value^24^. In order to preserve consistency and harmonization, this article will retain the initial symbols and their respective meanings. For better understanding, Figure 4 shows the description of the equations mentioned so far in this article.

**Figure 4:**
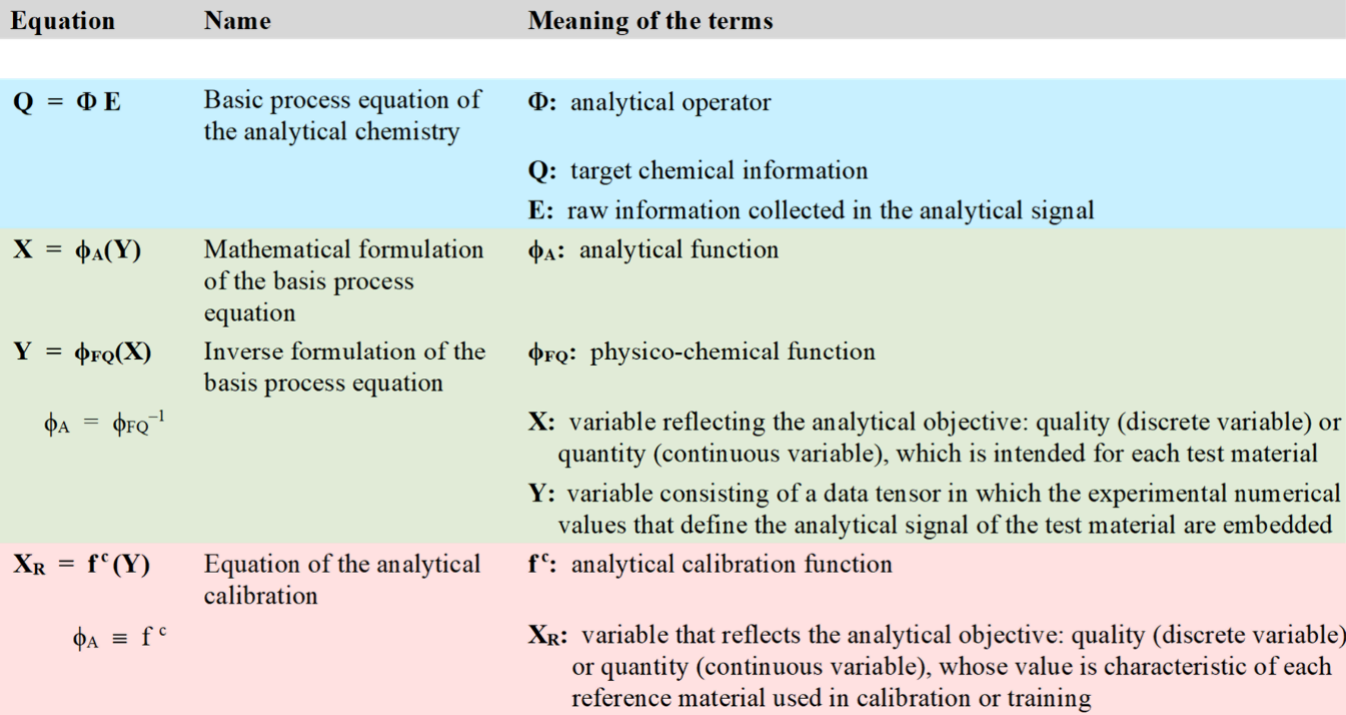
Description of the main equations and functions referred in this article.

The calibration functions most widely used by analytical chemists are univariate algebraic functions and, if feasible, linear functions, as they are very easy (and user-friendly) to apply and interpret. However, there are different ways of expressing and applying the multivariate calibration function **f** ^**c**^ with an increasing degree of mathematical complexity. These involve the following:^25–27^

(I) A single algebraic equation (linear or otherwise) defined by a set of numerical coefficients. This type is the essence of conventional least-squares regression methods. In the multivariable context, it is usually known as multiple linear regression (MLR). The simplest expression is the equation of a straight line, which determines a linear model on scalar data.
(II) A composite function resulting from applying two functions in series as a consequence of previously transforming the original data of **Y**-variables into a new system of **Z**-variables, called ‘latent variables’. In this way, two functions are developed during the training step: a first function **f** ^**c**^_**1**_ that transforms the original variables into latent variables, and a second function **f** ^**c**^_**2**_ that applies to the latent variables and determines the value of **X**-variable. Mathematically, this is shown in the eq 5:
$$\begin{array}{c} X_{R}=f^{c}(Y_{R})=({f^{c}}_{2}◦{f^{c}}_{1})(Y_{R})[(Y{)}_{R}\overset{{f^{c}}_{1}}{\to }Z\overset{{f^{c}}_{2}}{\to }X_{R}]\\ X_{R}={f^{c}}_{2}(Ζ);Z={f^{c}}_{1}(Y{)}_{R} \end{array}$$ 5

This strategy is typical of chemometric methods of multivariate regression involving latent variables, the simplest example of which is principal component regression (PCR). However, it is more usual to apply partial least-squares regression (PLS), where latent variables are calculated by considering both the **Y** and **X** variable data.

(III) A set of cross-linked functions that make up a resulting function of high mathematical complexity not explicit, which acts as a ‘black box’ and cannot be expressed simply. It is typical of artificial neural networks (ANN) and deep learning (DL).^28^ Its primary performance involves a set of processing units, referred to as nodes or neurons, all interconnected. As a whole, a function composed of three functions is applied: an input function **f** ^**c**^_**in**_ (it generates a net signal **N** as a weighted sum of the numerical data that make up the original **Y**-variable), an activation function **f** ^**c**^_**act**_ (it returns an activation value **A** by applying a screening to the net signal data), and an output function **f** ^**c**^_**out**_ (it provides the final result **X**).

In a simple way, the overall process can be written as the eq 6:

$$\begin{array}{c} X_{R}=f^{c}(Y_{R})=({f^{c}}_{\mathrm{out}}◦{f^{c}}_{\mathrm{act}}◦{f^{c}}_{\mathrm{in}})(Y_{R})[Y_{R}\overset{{f^{c}}_{in}}{\to }N\overset{{f^{c}}_{\mathrm{act}}}{\to }A\overset{{f^{c}}_{\mathrm{out}}}{\to }X_{R}]\\ X_{R}={f^{c}}_{\mathrm{out}}(Α);A={f^{c}}_{\mathrm{act}}(Ν);N={f^{c}}_{\mathrm{in}}(Y_{R}) \end{array}$$ 6

where **N** symbolizes the net signal generated by the input function, and **A** is the activation value resulting from applying the activation function.

## Example

To illustrate the role of the analytical operator throughout the different periods of analytical chemistry, we will consider the determination of the degree of unsaturation of vegetable oils as an example of an analytical target, which will constitute the term **Q.**

The first approach occurred in the early years of the 20th century, within the chemical age of analytical chemistry. Hasn Wijs proposed determining the rate of unsaturation in the fatty acid chains of triglycerides in a vegetable oil by adding iodine in glacial acetic acid and then volumetrically titrating the excess with thiosulfate, using starch as an indicator. The result, referred to as the iodine value, is expressed as the mass, in grams, of iodine consumed per 100 g of vegetable oil. The method is still in force,^29^ although some modifications have been proposed. The experimental information **E** is the volume of thiosulfate solution consumed in the titration, and its value is determined visually by color change (disappearance of a blue color). The analytical operator **Φ**, expressed mathematically, is the equation deduced from applying a mass balance based on the stoichiometry of the chemical reactions involved.

With the development of gas chromatography in the 1970s, already in the metrological age, the method for determining the mass composition profile of fatty acids in vegetable oils was consolidated. This is carried out by gas chromatography of fatty acid methyl esters (FAMEs), following a process of methyl transesterification of triglycerides. The method is still in place today and is widely used in vegetable oil quality control laboratories.^30^ From this composition, the iodine value is indirectly obtained. The experimental information **E** consists of the identity and measured area of each chromatographic peak corresponding to a particular FAME. Therefore, the mathematical form of the analytical operator **Φ** is an equation that multiplies the molecular mass value of iodine by the number of unsaturations in 100 g of vegetable oil, estimated from the composition of the major fatty acids.

Recently, different vibrational spectrometries (NIR, MIR, Raman) have provided several solutions for determining the iodine value. The experimental information **E** is now the spectrum acquired from the vegetable oil to be tested, using even portable equipment.^31^ Unfortunately, and for reasons beyond the scope of this article, none of these methods has yet been recognized as a standardized method by any relevant body, confirming that the data science age is not yet fully implemented. In this scenario, the analytical operator **Φ** is based on the application of a calibration function (model) obtained using a multivariate regression algorithm (e.g., PLS) that directly relates the iodine value (**X**-variable) to the data vector collecting the whole spectrum or a previously selected spectral region (**Y**-variable).

And moving into the world of fiction, in the age of artificial intelligence, it would be feasible for the iodine value to be determined just by ‘visualizing’ an image (experimental information **E**), acquired under specific conditions. This would be carried out using a suitable analytical device that does not require the establishment of mathematical models. It would work in the same way as the human brain when it is able to identify a certain person from a set of similar photographs. There are currently some options that could be applied, such as two-dimensional heteronuclear magnetic resonance (2D NMR HSQC), which can now be acquired using benchtop equipment. A precedent for this technology, still straddling the data science age and the artificial intelligence age, is the method that determines the iodine index from a hyperspectral image^32^ obtained from the fat. However, in this last study, establishing a mathematical quantification model applying multivariate statistics was still required.

## Final Remarks

Traditionally, R&D in analytical chemistry has focused on the **E** term of the basic process equation. When literature published in recognized journals are reviewed, besides those studies aimed at new applications using already known analytical systems, innovations are generally identified in sample preparation methods, advances in analytical instrumentation (including software), or development of new analytical devices. All of this leads to the **E** term and has generally focused on obtaining data derived from specific (or highly selective) analytical signals that require very simple data processing, so no novelties were needed in the term **Φ.**

Innovations that affect the term **Φ** in the basic equation are typically developed in the field of chemometrics. Such is the way with advances in data processing methods or algorithms, although too often these are based on simulated data because in many cases they are not proposed by analytical chemists, but by scientists devoted to data science. This block may also include proposals on univariate calibration methodologies,^33^ which were popular at the end of the last century but are now rarely reported. They were intended to correct or compensate for some of the common errors in univariate methods, such as those due to the sample introduction system or the matrix effect.

This divorce between researchers focused on **E** or **Φ** has led to a differentiation between ‘analytical chemists’ and ‘chemometricians’, as if the tasks and objectives of both groups were independent. This division is as perverse as it would be to consider analytical chemists on one side and those engaged in analytical chromatography on the other; something that would currently seem unheard of and beyond all logic. Currently, analytical researchers should maintain a simultaneous focus on both terms, as any advance in any methodology related to one of the terms of the basis equation has an impact on the minimum requirements of the other. This symbiotic approach to the focus of R&D activities involves a paradigm change for the analytical community and should not be avoided.

Finally, an open question is addressed to the readers: does it make sense that analytical chemistry today, in the era of data science and artificial intelligence, continues to rely primarily on traditional working methods and basic physical and mathematical processes developed in the 19th and 20th centuries? Analytical chemistry has changed in recent decades.^16^ On the one hand, analytical chemists are required to adapt to the new interdisciplinary reality,^12^ and on the other, we must communicate and disseminate what analytical chemistry is today in any forum where we have the opportunity, preferably in those where colleagues from other areas of chemistry are present. This spreading of the message is really important these days because old preconceptions about this discipline still persist. In essence, the well-known aphorism stated by C.N. Reilley in 1965 that “analytical chemistry is what analytical chemists do”^34^ still holds true.

## References

[1] Valcárcel M. (2016). Quo vadis,
analytical chemistry?. Anal. Bioanal. Chem..

[2] Lewenstam A., Zytkow J. M. (1987). Is analytical chemistry
an autonomous field of science?. Fresenius J.
Anal. Chem..

[3] Zytkow J. M., Lewenstam A. (1990). Analytical
chemistry – The science of many models. Fresenius J. Anal. Chem..

[4] Kissinger P. T. (1992). Analytical
Chemistry What is it? Who needs it? Why teach it?. Trends Anal. Chem..

[5] Honjo T. (2001). Analytical
Chemistry as methodology in modern pure and applied chemistry. Anal. Sci..

[6] Murray R. (1991). Analytical
chemistry: the science of chemical measurement.. Anal. Chem..

[7] Doerffel K. (1998). Analytical
science – A discipline between chemistry and metrology. Fresenius J. Anal. Chem..

[8] Danzer, K. Analytical Chemistry – Theoretical and Metrological Fundamentals; Springer-Verlag: Berlin Heidelberg, 2007; pp 1–5.

[9] Grasserbauer M. (1992). Competition
″Analytical chemistry – today’s definition and
interpretation″. Fresenius J. Anal. Chem..

[10] Kellner R. (1994). Education
of analytical chemists in Europe. Anal. Chem..

[11] Hibbert, B. Compendium of Terminology in Analytical Chemistry; The Royal Society of Chemistry: London, 2023; pp 2.

[12] Síma J. (2016). Modern analytical
chemistry in the contemporary world. Cult. Stud.
Sci. Educ..

[13] Kowalski B. R. (1981). Analytical
chemistry as an information science. Trends
Anal. Chem..

[14] Booksh K. S., Kowalski B. R. (1994). Theory of analytical chemistry. Anal. Chem..

[15] Karayannis M. I., Efstathiou C. E. (2012). Significant steps in the evolution of analytical chemistry
– Is the today’s analytical chemistry only chemistry?. Talanta.

[16] Adams F., Adriaens M. (2020). The metamorphosis of analytical chemistry. Anal. Bioanal. Chem..

[17] Szymanska E. (2018). Modern data
science for analytical chemical data – A comprehensive review. Anal. Chim. Acta.

[18] Galuszka A., Migaszewski Z., Namiesnik J. (2013). The 12 principles
of green analytical
chemistry and the SIGNIFICANCE mnemonic of green analytical practices. Trends Anal. Chem..

[19] Kadis R. L. (2013). Measurement
of an analytical signal or measurement of the content of an analyte?. J. Anal. Chem..

[20] Currie L. A. (1995). Nomenclature
in evaluation of analytical methods including detection and quantification
capabilities. Pure Appl. Chem..

[21] Cuadros Rodríguez, L. ; González Casado, A. ; Ruiz Samblás, C. ; Bagur González, M. G. Evolution of the quality concept in analytical laboratories. In Encyclopedia of Analytical Chemistry – Online; Meyers, R. A. , Ed.; John Wiley & Sons: Hoboken, 2017.

[22] Cuadros
Rodríguez L., Jiménez Carvelo A. M., Fernández
Ramos M. D. (2021). Multivariate thinking for optical microfluidic analytical
devices – A tutorial review. Microchem.
J..

[23] Sun W., Braatz R. D. (2020). Opportunities in
tensorial data analytics for chemical
and biological manufacturing processes. Comput.
Chem. Eng..

[24] Hibbert D. B. (2016). Vocabulary
of Concepts and Terms in Chemometrics. Pure
Appl. Chem..

[25] Sanchez E., Kowalski B. R. (1988). Tensorial calibration
– I. First-order calibration. J. Chemom..

[26] Brereton R. G. (2000). Introduction
to multivariate calibration in analytical chemistry. Analyst.

[27] Bro R. (2003). Multivariate
calibration – What is in chemometrics for the analytical chemist?. Anal. Chim. Acta.

[28] Andrade-Garda, J. M. ; Gestal-Pose, M. ; Cedrón-Santaeufemia, F. A. ; Dorado-de-la-Calle, J. ; Gómez-Carracedo, M. P. Multivariate Regression using Artificial Neural Networks and Support Vector Machines. In Basic Chemometric Techniques in Atomic Spectroscopy: Andrade-Garda, J. M. , Ed.; 2nd ed; The Royal Society of Chemistry: Cambridge, 2013; pp 348–397.

[29] ASTM D5554–15 Standard test method for determination of the iodine value of fats and oils; ASTM International: West Conshohocken, PA, 2021.

[30] ISO 12966–4 Animal and vegetable fats and oils – Gas chromatography of fatty acid methyl esters. Part 4: Determination by capillary gas chromatography; International Organization for Standardization: Geneva, 2015.

[31] Yan H., Zhang J., Gao J., Huang Y., Xiong Y., Min S. (2018). Towards improvement in prediction of iodine value in edible oil system
based on chemometric analysis of portable vibrational spectroscopic
data. Sci. Rep..

[32] Kucha C. T., Liu L., Ngadi M., Claude G. (2021). Hyperspectral imaging and chemometrics
as a non-invasive tool to discriminate and analyze iodine value of
pork fat. Food Control.

[33] Kóscielniak, P. Calibration in Analytical Science – Methods and Procedures; Wiley-VCH GmbH: Weinheim, 2023.

[34] Murray R. (1994). Analytical
chemistry is what analytical chemists do. Anal.
Chem..

